# Cobalt(II) Complexes with *N*,*N*,*N*-Scorpionates and Bidentate Ligands: Comparison of Hydrotris(3,5-dimethylpyrazol-1-yl)borate Tp* vs. Phenyltris(4,4-dimethyloxazolin-2-yl)borate To^M^ to Control the Structural Properties and Reactivities of Cobalt Centers

**DOI:** 10.3390/molecules23061466

**Published:** 2018-06-16

**Authors:** Toshiki Nishiura, Takahiro Uramoto, Yuichiro Takiyama, Jun Nakazawa, Shiro Hikichi

**Affiliations:** Department of Material and Life Chemistry, Faculty of Engineering, Kanagawa University, 3-27-1 Rokkakubashi, Kanagawa-ku, Yokohama 221-8686, Japan; r201670152kn@jindai.jp (T.N.); r201304066bf@jindai.jp (T.U.); yuichiro0411@gmail.com (Y.T.)

**Keywords:** scorpionate ligand, borate, cobalt, oxidation

## Abstract

Scorpionate ligands Tp* (hydrotris(3,5-dimethylpyrazol-1-yl)borate) and To^M^ (tris(4,4-dimethyloxazolin-2-yl)phenylborate) complexes of cobalt(II) with bidentate ligands were synthesized. Both Tp* and To^M^ coordinate to cobalt(II) in a tridentate fashion when the bidentate ligand is the less hindered acetylacetonate. In crystal structures, the geometry of cobalt(II) supported by the N_3_O_2_ donor set in the Tp* complex is a square-pyramid, whereas that in the To^M^ complex is close to a trigonal-bipyramid. Both Tp*- and To^M^-acac complexes exhibit solvatochromic behavior, although the changing structural equilibria of these complexes in MeCN are quite different. In the bis(1-methylimidazol-2-yl)methylphenylborate (L^Ph^) complexes, Tp* retains the tridentate (*к*^3^) mode, whereas To^M^ functions as the bidentate (*к*^2^) ligand, giving the tetrahedral cobalt(II) complex. The bowl-shaped cavity derived from the six methyl groups on To^M^ lead to susceptibility to the bulkiness of the opposite bidentate ligand. The entitled scorpionate compounds mediate hydrocarbon oxidation with organic peroxides. Allylic oxidation of cyclohexene occurs mainly on the reaction with *tert*-butyl hydroperoxide (TBHP), although the catalytic efficiency of the scorpionate ligand complexes is lower than that of Co(OAc)_2_ and Co(acac)_2_. On cyclohexane oxidation with *meta*-chloroperbenzoic acid (*m*CPBA), both To^M^ and Tp* complexes function as catalysts for hydroxylation. The higher electron-donating To^M^ complexes show faster initial reaction rates compared to the corresponding Tp* complexes.

## 1. Introduction

Facially capping tridentate ligands are utilized to synthesize tetra-, penta-, and hexa-coordinated transition metal complexes. The appropriate molecular design of the ligands controls the coordination geometry of the metal center. For example, bulky shading substituent groups on ligands stabilize low-coordination numbers of the metal centers of the resulting complexes. A family of scorpionates—a nickname for chelating borate ligands—is extensively employed for a variety of coordination compounds due to its molecular design versatility [1,2]. A prototype of this family is hydrotris(pyrazol-1-yl)borate, [HB(pz)_3_]^‒^ (Tp), which is composed of pyrazole. The third, fourth, and fifth positions of pyrazole can be modified by introducing various groups, and the resulting substituted pyrazoles are also applied to borate ligands such as [HB(pz^R^)_3_]^‒^ (Tp^R^; R denotes the substituent groups on pyrazole). Hydrotris(3,5-dimethylpyrazol-1-yl)borate, called Tp* (also called Tp^Me2^), is classified as “the first generation of scorpionates” along with the nonsubstituted Tp. Its moderate bulkiness, derived from the methyl groups, allows it to form both homo- and heteroleptic complexes (e.g., [M(*к*^3^-Tp*)_2_] and [M(*к*^3^-Tp*)L*_x_*]; L denotes ligands other than Tp*), in the latter of which the metal centers have various coordination numbers. Therefore, various metal complexes with Tp* have been investigated extensively so far [1,2].

To date, novel scorpionate-type borate ligands with various non-pyrazolyl donors such as azoles [3,4,5,6], thioethers [7], phosphines [8], and N-heterocyclic carbenes [9] have been developed. Among them, tris(oxazolin-2-yl)borates, called To^R^ (R indicates substituent groups on the fourth and fifth positions of oxazoline), are attractive because various substituted oxazoline derivatives are available. The first To^R^ is tris(4,4-dimethyloxazolin-2-yl)phenylborate (To^M^), of which six methyl groups form a bowl-shaped cavity surrounding the metal center on the resulting metal complexes [5]. To date, tetra-, penta-, and hexa-coordinated heteroleptic complexes with To^M^ have been reported [5,10,11,12,13,14].

Structural and electronic properties of Tp^R^ and To^R^ have been compared on tricarbonyl complexes of group VII metals (rhenium and manganese) [12,13]. Steric bulkiness of To^M^ is higher than that of Tp* estimated by solid angles derived from the crystal structures. The local structures of the hexa-coordinated metal centers of [M^I^(*к*^3^-Tp*)(CO)_3_] and [M^I^(*к*^3^-To^M^)(CO)_3_] are, however, very close, because no steric repulsions exist between the metal-surrounding methyl groups on the scorpionates and the liner diatomic CO ligands. The electron-donating ability of To^M^ is higher than that of Tp* estimated from ν_CO_ of IR measurement. From electrochemical investigations, however, the donating ability of Tp* is higher than that of To^M^ according to the order of the oxidation potentials, [M^I^(*к*^3^-Tp*)(CO)_3_] < [M^I^(*к*^3^-To^M^)(CO)_3_]. Such different interpretations arise from differences in the oxidation state. The former estimation based on IR data is attributed to the single oxidation state of the metal centers, while the latter results from changing the oxidation state of the metal centers on the rigid pseudo-octahedral complexes [12].

We have also examined the structural properties of Tp* and To^M^ in the crystal structures of the pseudo-tetrahedral nickel(II)-chloride complexes [Ni^II^(*к*^3^-Tp*)Cl] and [Ni^II^(*к*^3^-To^M^)Cl]. The Tp* complex is very close to the symmetric structure of C_3v_, as evidenced by an almost linear arrangement of Cl-Ni---B_Tp_. In the To^M^ complex, however, an arrangement of Cl-Ni---B_To_ deviates from a straight line. Therefore, To^M^ does not work as a “tetrahedral enforcer,” although solid angle analysis [15] of the nickel-chloride complexes reveals that To^M^ is bulkier than Tp*. Six methyl groups of To^M^ surrounding a metal center form a bowl-shaped cavity, but not a deep one. Therefore, the arrangement of a monodentate ligand is relatively flexible [14].

In this work, we investigated coordination behaviors of Tp* and To^M^ toward cobalt(II) with bidentate ligands (D–D). Cobalt(II) shows a flexible coordination structure. Varied structural and electronic properties of the bidentate ligands reflect on the molecular structures and reactivities of the resulting mixed ligand complexes, [Co^II^(scorpionate)(D–D)]. We focused on the catalytic performance of the synthesized mixed ligand complexes toward alkene oxidation with *tert*-BuOOH (TBHP) and alkane oxidation with *meta*-chloroperbenzoic acid (*m*CPBA), as the estimation of the functions depends on the scorpionate ligands. These aspects provide useful information for the design of functional metallocomplexes with appropriate ligands.

## 2. Results and Discussion

### 2.1. Synthesis and Characterization of Mixed Ligand Complexes

**Scheme 1:**
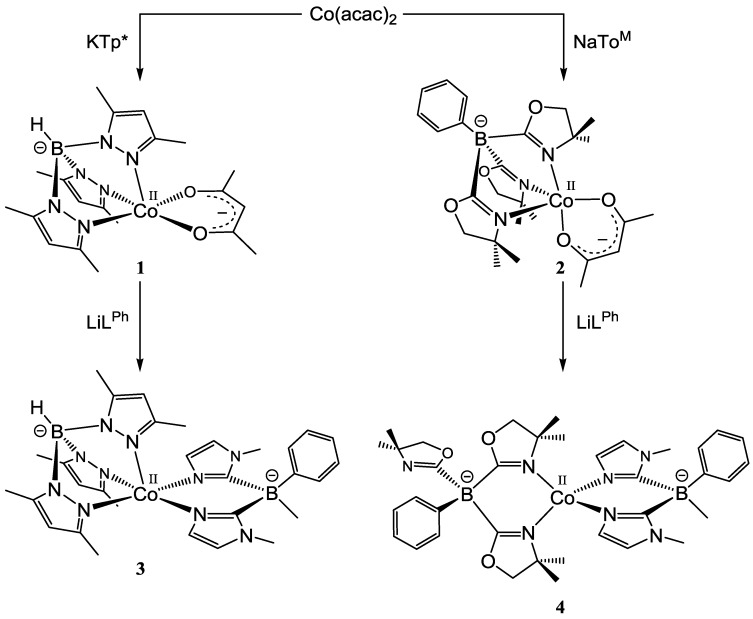
Synthesis of mixed ligand complexes **1**–**4**.

In this work, two kinds of −1-charged bidentate ligands were employed. One was acetylacetonate (acac) as an oxygen-donating ligand, and the other was methylbis(1-methylimidazol-2-yl)phenylborate (L^Ph^; [B(Im*^N^*^-Me^)_2_MePh]^‒^) as a nitrogen-donating ligand [16,17,18,19]. These ligands exhibited different structural properties. In acac, no steric hindrance exists around the oxygen donors. In contrast, the nitrogen donors of L^Ph^ are incorporated in the five-member imidazolyl rings and the fourth-positioned sp^2^-C‒H group overhangs. Arrangements of two donor atoms of these ligands are also different. Acac has a planar configuration, and O‒M‒O bite angles of the formed complexes are almost fixed to right angles. On the other hand, two imidazolyl rings of L^Ph^ bind to the sp^3^-boron center and dihedral angles of the imidazolyl rings and N‒M‒N bite angles are somewhat flexible [17]. Therefore, we examined the combination of the scorpionates (Tp* or To^M^) and these bidentate ligands (acac or L^Ph^) in order to clarify the correlations between the bulkiness and the coordination behavior of Tp* vs. To^M^ (Scheme 1).

#### 2.1.1. Acetylacetonato Complexes [Co^II^(Tp*)(acac)] (**1**) and [Co^II^(To^M^)(acac)] (**2**)

The acac complexes were synthesized by ligand metathesis reactions between Co^II^(acac)_2_ and the alkaline metal salts of the scorpionates (KTp* for **1** and NaTo^M^ for **2**) under anhydrous conditions. Adding the THF solution of KTp* or NaTo^M^ to the THF solution of Co^II^(acac)_2_ yielded the desired complexes [Co^II^(Tp*)(acac)] (**1**) and [Co^II^(To^M^)(acac)] (**2**), respectively. It is known that homoleptic complexes [Co^II^(*к*^3^-Tp*)_2_] [20] and [Co^II^(*к*^2^-To^M^)_2_] [10] are formed as byproducts during the reaction of scorpionate ligands and Co^II^X_2_ (X = Cl, OAc, NO_3_, etc.). In the case of To^M^, a protonated ligand complex such as [Co^II^Cl_2_(HTo^M^)] is also formed in the presence of proton sources [10]. In the present system, yields of these byproducts were small.

**Figure 1:**
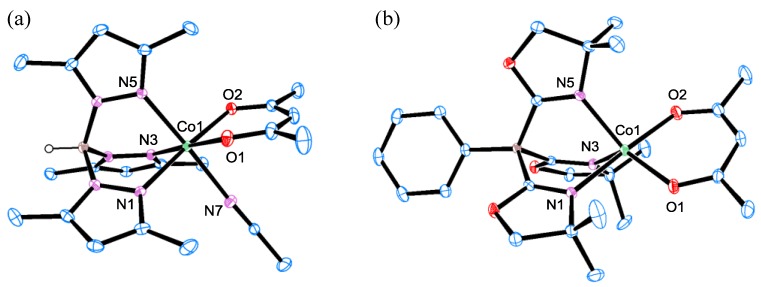
Molecular structures of (**a**) **1**·MeCN and (**b**) **2**. All hydrogen atoms except B–H moiety are omitted for clarity; thermal ellipsoids are set at 30% probability. Selected bond lengths [Å] and angles [°]: for **1**·MeCN; Co1-N1 2.153 (1), Co1-N3 2.101 (1), Co1-N5 2.124 (1), Co1-O1 2.055 (1), Co1-O2 2.054 (1), N1-Co1-N3 88.05 (5), N1-Co1-N5 87.01 (5), N1-Co1-N7 90.76 (5), N1-Co1-O1 91.47 (5), N1-Co1-O2 176.67 (5), N3-Co1-N5 86.33 (5), N3-Co1-N7 92.80 (5), N3-Co1-O1 178.72 (5), N3-Co1-O2 93.03 (5), N5-Co1-N7 177.63 (5), N5-Co1-O1 94.83 (5), N5-Co1-O2 96.20 (5), O1-Co1-O2 87.38 (5). For **2**; Co1-N1 2.177 (1), Co1-N2 2.082 (1), Co1-N3 2.063 (1), Co1-O4 2.005 (1), Co1-O5 2.028 (1), N1-Co1-N2 84.94 (4), N1-Co1-N3 87.60 (4), N1-Co1-O4 87.07 (4), N1-Co1-O5 172.67 (5), N2-Co1-N3 90.83 (4), N2-Co1-O4 136.48 (4), N2-Co1-O5 94.46 (5), N3-Co1-O4 131.50 (5), N3-Co1-O5 99.55 (5), O4-Co1-O5 86.98 (4).

Both synthesized acac complexes were fully characterized by spectroscopy (IR, ^1^H-NMR, UV-vis) and X-ray crystallography. Molecular structures of an MeCN adduct of **1** and **2** are shown in Figure 1. Geometries of the five-coordinated cobalt centers supported by tridentate scorpionates and bidentate acac ligands are a square-pyramid in **1** (when the coordinating MeCN molecule is omitted) and a distorted trigonal-bipyramid in **2**. In Tp* complex **1**, both oxygen donors of acac were located on equatorial positions. In To^M^ complex **2**, however, one of the two oxygen donors from acac sat on the trigonal plane and another was on the axial position. Such difference of locations of the acac ligands in the static solid state arose from steric hindrance derived by cobalt-surrounding methyl groups on the azole rings of the scorpionates. In **2**, steric repulsion between two equatorial 4,4-dimethyl oxazoline groups of To^M^ and a virtual equatorial acac ligand would occur. Similar structural variations depending on the bulkiness of substituent groups of Tp^R^ are also observed in [Co^II^(*к*^3^-Tp^3R^)( *к*^3^-acac)] (3R denotes substituent groups on the third position of the pyrazolyl rings) [21,22,23,24]. When 3R = Ph, the geometry of the cobalt center is trigonal-bipyramid [22]. In contrast, the diphenylmethyl analogue (i.e., 3R = CHPh_2_) has a square-pyramidal cobalt center [23]. Space around the metal center of the Tp^3CHPh^^2^ complex is close to that of the Tp* complex because three tertiary sp^3^-C–H groups surround the metal center.

**Figure 2:**
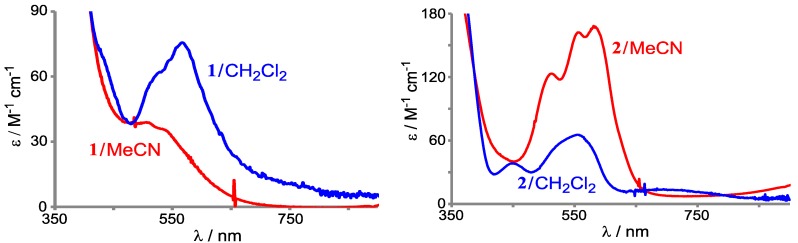
UV-vis spectra of CH_2_Cl_2_ and MeCN solutions of **1** (**left**) and **2** (**right**).

**Scheme 2:**
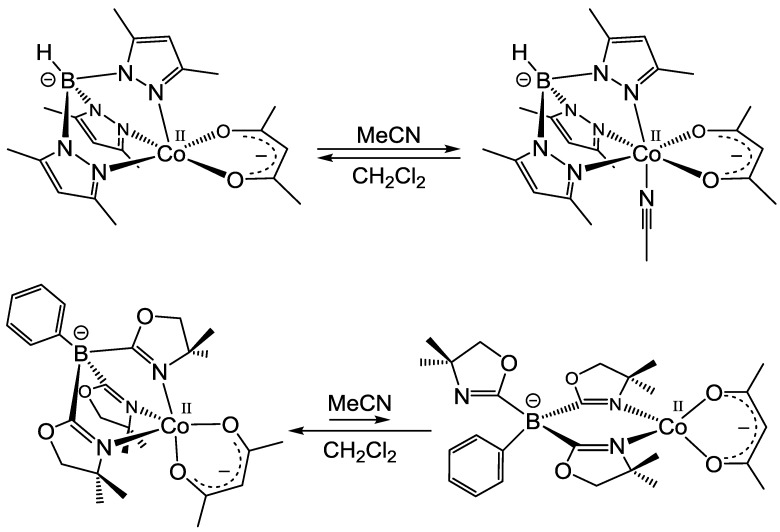
Solvent-dependent equilibrium of structural change of **1** (**top**) and **2** (**bottom**).

Both **1** and **2** exhibited solvatochromic behavior. UV-vis spectra of CH_2_Cl_2_ and MeCN solutions of **1** and **2** showed different patterns (Figure 2). For Tp* complex **1**, its CH_2_Cl_2_ solution showed moderate-intensity (*ε* ≈ 80 M^–1^ cm^–1^) absorption around the 500–570 nm region, whereas the MeCN solution revealed weakened absorption (*ε* ≈ 40 M^–1^ cm^–1^) around the 480–550 nm region. This changing spectral behavior was similar to that observed for the previously reported Tp^Me3^ ligand complex [Co^II^(*к*^3^-NO_3_)(*к*^3^-Tp^Me3^)] [25]. In the MeCN solution, these Tp^Me2,X^ complexes turned to the corresponding solvated hexa-coordinated Co(II) species as found in the crystal structure (see Figure 1 and Scheme 2). In contrast, To^M^ complex **2** showed different behavior. The CH_2_Cl_2_ solution of **2** exhibited weak to moderate-intensity absorption at 450 nm (*ε* ≈ 40 M^–1^ cm^–1^) and 555 nm (*ε* ≈ 65 M^–1^ cm^–1^). When the solvent was changed to MeCN, multiple absorption with increased intensity could be observed at 513 nm (*ε* ≈ 120 M^–1^ cm^–1^), 557 nm (*ε* ≈ 160 M^–1^ cm^–1^), and 584 nm (*ε* ≈ 170 M^–1^ cm^–1^). This spectral pattern was similar to that attributed to four-coordinated tetrahedral cobalt(II) species rather than the six-coordinated species as found for the above-mentioned MeCN solutions of the Tp* complexes including **1**. A plausible explanation for this is coordination-dissociation equilibrium of one of three oxazolynyl nitrogen donors of To^M^ induced by the action of MeCN (Scheme 2). The spectral patterns of ^1^H-NMR signals of CDCl_3_ and *d*_3_-MeCN solutions of **2**, however, were not so different (see Supplementary Figures S1 and S2). Therefore, the putative tetrahedral species resulting from partial dissociation of To^M^ might not be so favored.

#### 2.1.2. Bis(1-methylimidazol-2-yl)borato Complexes [Co^II^(Tp*)(L^Ph^)] (**3**), [Co^II^(To^M^)(L^Ph^)] (**4**) and [Co^II^(L^Ph^)_2_] (**5**)

Bis(1-methylimidazol-2-yl)methylphenylborate L^Ph^ ([B(Im*^N^*^-Me^)_2_MePh]^‒^) [16,17,18,19] was also examined as another monoanionic bidentate ligand. The reaction of LiL^Ph^ and **1** or **2** resulted in selective ligand exchange from acac to L^Ph^ to give the corresponding Tp* (**3**) and To^M^ (**4**) complexes, respectively. In these L^Ph^ complexes, the coordination behavior of Tp* and To^M^ was quite different.

Tp* complex **3** had already been reported as an analogue of a penta-coordinated iron(II) complex [Fe(Tp*)(L^Ph^)], which showed O_2_-binding capability to yield the corresponding mononuclear iron(III)-superoxo compound [18]. In the original report, **3** had been obtained from a cobalt(II)-acetato complex, [Co(Tp*)(*к*^2^-OAc)] [25]. As described in the previous report, the molecular structure of **3** had been revealed by X-ray crystallography. The geometry of the cobalt(II) center of **3** is very close to a square-pyramid. Two nitrogen donors from L^Ph^ sit on the equatorial position, with an almost right angle of its N–Co–N bite angle (88.4°). Two imidazolyl rings of L^Ph^ bend each other (estimated dihedral angle was 142°), and the resulting L^Ph^ meshed with threefold symmetrically arranged pyrazoles of Tp* due to steric repulsion between the C–H groups of the fourth position of the imidazoles of L^Ph^ and the methyl groups attached on the third position of the pyrazoles of Tp*.

**Figure 3:**
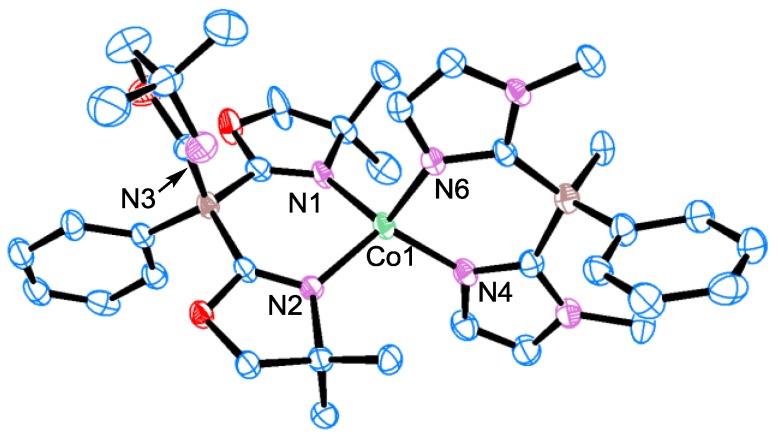
Molecular structure of **4**. All hydrogen atoms are omitted for clarity; thermal ellipsoids are set at 30% probability. Selected bond lengths [Å] and angles [°]: Co1-N1 2.004 (3), Co1-N2 2.000 (3), Co1-N4 1.989 (4), Co1-N6 1.972 (3), N1-Co1-N2 97.0 (1), N1-Co1-N4 121.9 (1), N1-Co1-N6 110.6 (1), N2-Co1-N4 115.4 (1), N2-Co1-N6 115.3 (1), N4-Co1-N6 97.7 (1).

**Scheme 3:**
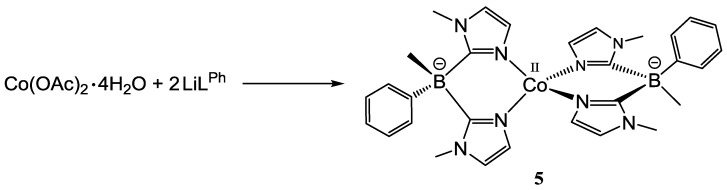
Synthesis of [Co^II^(L^Ph^)_2_] (**5**).

In contrast, To^M^ complex **4** was a tetrahedral cobalt(II) complex with bidentate To^M^ and L^Ph^ ligands, as shown in Figure 3. Two imidazolyl rings of L^Ph^ in **4** were located almost coplanar (as estimated dihedral angle was 176°), and an N–Co–N bite angle (97.7°) was larger than a right angle. Because of the steric hindrance derived from the methyl groups on the oxazoline rings of To^M^, it was impossible to form a penta-coordinated cobalt(II) complex with *к*^3^-To^M^ and *к*^2^-L^Ph^. One of three oxazolynyl groups of To^M^ in **2** was left from the cobalt center through the ligand substitution from acac to L^Ph^ such that the bidentate To^M^ was observed in a homoleptic complex, [Co^II^(*к*^2^-To^M^)_2_] [10]. Similarities of the geometry of the cobalt(II) centers and the coordination mode of To^M^ were also reflected on spectroscopic properties. IR spectra of the solid samples of **4** (Supplementary Figure S3c) and [Co^II^(*к*^2^-To^M^)_2_] exhibited characteristic peaks around 1600 and 1575 cm^−1^ and 1603 and 1554 cm^−1^, respectively. The bands around 1600 cm^−1^ would be attributed to *ν*C–N of the noncoordinating oxazolynyl groups of *к*^2^-To^M^. UV-vis spectra of CH_2_Cl_2_ solutions showed intense band characteristics to the tetrahedral cobalt(II) d–d transition at *λ*max = 525 (*ε* ≈ 440 M^–1^ cm^–1^) and 554 nm (*ε* ≈ 580 M^–1^ cm^–1^) for **4** (Supplementary Figure S4) and *λ*max = 562 (*ε* = 539 M^−1^ cm^−1^) and 576 nm (*ε* = 552 M^−1^ cm^−1^) for [Co^II^(*k*^2^-To^M^)_2_]. The observed blue shift of the d–d transition bands from those of [Co^II^(*k*^2^-To^M^)_2_] to **4** might imply that the strength of the ligand fields is L^Ph^ > To^M^. In fact, a homoleptic L^Ph^ complex [Co^II^(L^Ph^)_2_] (**5**; a preliminary result of single crystal X-ray analysis is provided as Supplementary Figure S5), which was synthesized by the reaction of Co(OAc)_2_ and two equiv. of L^Ph^ (Scheme 3), exhibited the tetrahedral cobalt(II) d–d transition at *λ*max = 520 (*ε* ≈ 300 M^–1^ cm^–1^) and 548 nm (*ε* ≈ 420 M^–1^ cm^–1^) (Supplementary Figure S4).

#### 2.1.3. Oxidation Potentials

As we have reported previously, Tp*-L^Ph^ complex **3** exhibits reversible O_2_ sorption/desorption ability at low temperature [18]. In this work, we checked the O_2_ binding capability of acac complexes **1** and **2** and To^M^-L^Ph^ complex **4** toward dioxygen. However, these cobalt(II) complexes were inert toward dioxygen even at −80 °C. The homoleptic L^Ph^ complex **5** was also inactive. Therefore, oxidation potentials of the cobalt(II) centers of **1**, **2**, **4**, and **5** were estimated by CV and compared with the previously reported cobalt(II)/(III) potential of **3** (Supplementary Figure S6).

Among the examined complexes, To^M^-L^Ph^ complex **4** exhibited the lowest cobalt(II/III) potential. The order of the oxidation potentials was **4** (0.49 V) < **2** (0.77 V) < **1** (1.15 V) < **5** (1.18 V). However, the observed potential of the MeCN solution of **4** was still higher than the reported value for **3** (0.18 V). Notably, a dihydrobis(pyrazolyl)borate (Bp) analogue of **3**, that is, [Co^II^(Tp*)(Bp)], showed reversible O_2_ sorption/desorption ability at low temperature, although its oxidation potential was higher (0.52 V) than that of **4** [26]. No O_2_ binding ability of **4** could be attributed to a structural reason (i.e., it was impossible to form a desired six-coordinated cobalt(III)-O_2_ adduct species). In the case of acac and *к*^3^-scorpionate complexes **1** and **2**, the difference of the potentials can be correlated with the extent of the electron donation from the scorpionate ligands. The trend of the electron-donating abilities of To^M^ vs. Tp* in the entitled acac complexes of cobalt(II) is inconsistent with that in the previously reported [M^I^(*к*^3^-scorpionate)(CO)_3_] [12]. In the present cobalt complexes, the observed cyclic voltammograms were irreversible. Therefore, structural factors should be considerable.

### 2.2. Oxidation Catalysis

To date, some cobalt compounds, including Tp^R^ [27,28] and To^M^ [10] complexes, have been employed as catalysts for hydrocarbon oxygenation with organic peroxides (ROOH) such as *tert*-butyl hydroperoxide (TBHP) [28,29,30,31,32,33,34,35,36] and *meta*-chloroperbenzoic acid (*m*CPBA) [10,37,38,39,40,41,42,43]. Therefore, we explored catalysis of the entitled mixed ligand complexes toward alkene oxidation with TBHP and alkane oxidation with *m*CPBA as indicated below. We also examined catalytic oxidation of alkenes and alkanes with H_2_O_2_. But the entitled mixed ligands complexes **1**–**4** were inactive in the H_2_O_2_ system.

#### 2.2.1. Oxidation of Alkene with TBHP

We reported on Tp-based immobilized cobalt complex catalysts and their activity toward cyclohexene oxidation with TBHP [28]. Cyclohexane is a valuable substrate as a probe to gain insights into the character of the active oxidant and reaction mechanism. When an oxidant reacting with cyclohexene has a radical character, allylic oxidation rather epoxidation of the C=C moiety is favored. Products derived from the allylic oxidation are allylic alcohol (***A***), ketone (***K***), and peroxide (***P***). Formation of ***P*** indicates involvement of alkylperoxyl radical (*tert*-BuOO·), which is formed by degradation of TBHP through the Habor–Weiss mechanism and/or homolysis of an M–O bond of a putative metal–alkylperoxo complex [36,44]. In our previous work, catalytic activity of the cobalt(II) compounds, including a genuine cobalt(II) acetate, tetrahedral cobalt(II) complex [Co^II^Br(allyl-Tp^CF3^)] (allyl-Tp^CF3^ denotes allyltris(3-trifluoromethyl-1-pyrazol-1-yl)borate), and mesoporous silica-supported Tp^CF3^Co complexes, was examined without any additive, and the activity of Co^II^(OAc)_2_·4H_2_O and [Co^II^Br(allyl-Tp^CF3^)] under homogeneous conditions was lower than that of the immobilized complexes under heterogeneous reactions [28].

In this study, small amounts of triethylamine (5 equiv. of cobalt) were added. As shown in Table 1 and Table 2, the major product was ***P*** in all cases, and the yields of ***E*** were very low. When the remaining TBHP in the reaction mixture was quenched by PPh_3_ prior to the product analysis, the yields of the allylic oxidation products ***A*** and ***K*** were decreased, although the major product was ***P*** (Supplementary Figure S8). These trends suggested that the reactions proceeded through a radical mechanism. It is known that oxidations of aliphatic C–H groups with H_2_O_2_ through radical mechanisms result in generation of the corresponding alkylhydroperoxides, and the resulting alkylhydroperoxides are converted to alcohols when the reaction mixtures are treated with PPh_3_ quencher prior to analysis [43]. In our system, the absence of an increase of ***A*** suggested that cyclohexenylhydroperoxide did not form. The activities of the entitled mixed ligand complexes **1**–**4** and the homoleptic complexes of L^Ph^ (**5**) and Tp* ([Co^II^(Tp*)_2_] (**6**) [20]) were lower than those of Co^II^(OAc)_2_·4H_2_O and Co^II^(acac)_2_·2H_2_O. The order of the activities (estimated by turnover numbers (TONs)) of the mixed ligand complexes **1**–**4** was **2** > **4** > **1** > **3** at ambient temperature and **4** ≈ **2** > **1** > **3** at 60 °C. In terms of correlation between the activities and the scorpionates and bidentate ligands, To^M^ was better than Tp* and acac was better than L^Ph^. In the Tp* complexes, the order of the activities was **6** > **1** > **3** at both ambient temperature and 60 °C. In the L^Ph^ complexes, the order of activities was **4** > **5** > **3** at ambient temperature and **4** > **3** > **5** at 60 °C. The order of Co(II)/(III) oxidation potentials was **5** (1.18 V) ≈ **1** (1.15 V) > **2** (0.77 V) > **4** (0.49 V) > **6** (0.36 V) > **3** (0.18 V), and that was inconsistent with the order of the activities. Coverage of the cobalt centers by the scorpionate ligands hindered the access of TBHP, and that might be a reason for lower activity compared to the less hindered cobalt(II) compounds such as Co^II^(OAc)_2_·4H_2_O and Co^II^(acac)_2_·2H_2_O.

It is known that Co^II^(acac)_2_ reacts with excess TBHP in the presence of N-donating L ligands such as pyridine or 1-methyimidazole to yield the corresponding cobalt(III)-OO*t*Bu complex [Co^III^(OO*t*Bu)(acac)_2_(L)] [29,30]. In contrast, relatively hindered Tp^R^ ligand hydrotris(3-alkyl-5-*iso*propylpyrazol-1-yl)borates such as Tp*^i^*^Pr2^ (when 3-alkyl = *iso*propyl) and Tp*^t^*^Bu,*i*Pr^ (when 3-alkyl = *tert*-butyl) stabilize the corresponding alkylperoxo complexes of cobalt(II), [Co^II^(OO*t*Bu)(Tp^R^)] [45]. These results clearly indicate that redox properties of the cobalt centers depend on the supporting ligands. The redox property of the cobalt center tuned by the ligands would affect not only the formation of alkylperoxo complex through direct interaction between cobalt and TBHP, but also outer-sphere electron transfer to cause TBHP degradation. Therefore, both steric and electronic properties of the scorpionates and bidentate ligands affect the catalysis of the cobalt centers.

#### 2.2.2. Oxidation of Alkane with *m*CPBA

Sadow and coworkers demonstrated that an acetate complex of cobalt(II) with To^M^, [Co^II^(To^M^)(OAc)], functions as an efficient catalyst precursor for cyclohexane hydroxylation with *m*CPBA [10]. In contrast, we reported that a hydroxo complex of cobalt(II) with Tp*, [(Co^II^Tp*)_2_(*μ*-OH)_2_], did not function as a catalyst precursor for alkane oxidation with *m*CPBA when the ratio of cobalt(II) to *m*CPBA to cyclohexane was 1:50:2500 [46]. However, the examined conditions (ratio of cobalt(II) to *m*CPBA to cyclohexane, solvent, temperature) for the To^M^ and Tp* complexes were different. In this work, the applicability of heteroleptic and homoleptic complexes **1**–**6** toward *m*CPBA-based alkane oxidation system was examined under the same conditions.

**Figure 4:**
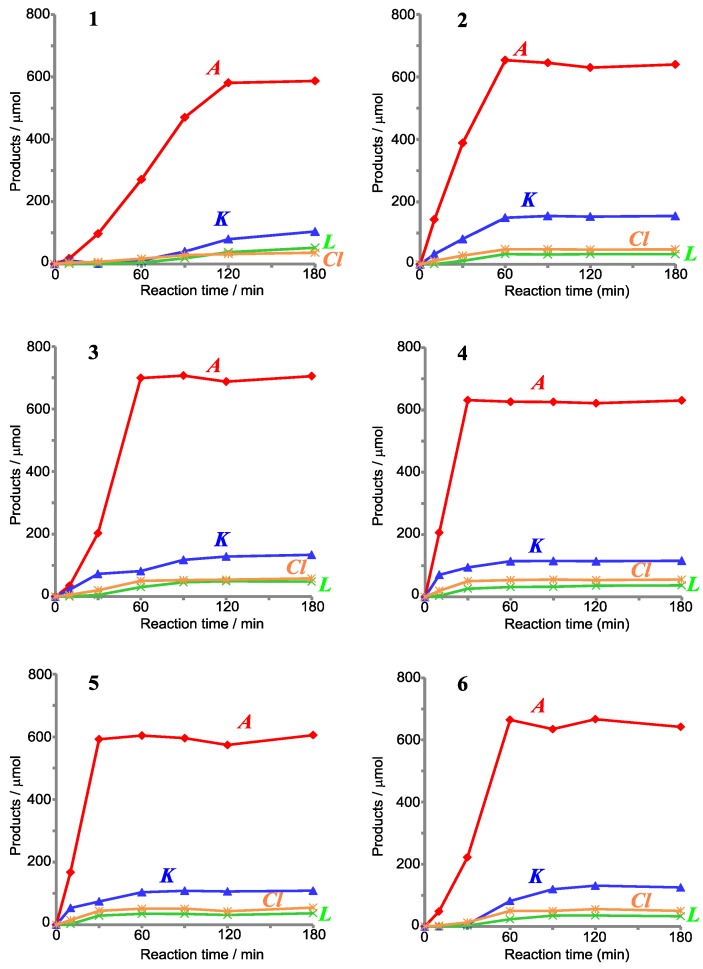
Time course of cyclohexane oxidation with *m*CPBA mediated by **1**–**6**.

All the examined complexes **1**–**6** exhibited activities, and their alcohol selectivity (***A***/***K*** + ***L***) was higher than that of the precursors (Co^II^(OAc)_2_·4H_2_O and Co^II^(acac)_2_·2H_2_O) as found in Table 3. The order of TONs of the mixed-ligand complexes was **3** > **2** > **4** > **1**. As shown in Figure 4, the order of initial reaction rates was **4** > **2** ≈ **3** > **1**. In the comparison of homoleptic complexes **5** and **6**, the order of the initial reaction rates was **5** > **6,** although total TONs of **5** and **6** were comparable. These trends of initial reaction rates support that the electron-donating ability of Tp* is lower than that of To^M^. Although the reaction mediated by **3** proceeded slowly, the lifetime of the catalyst derived from **3** was longer than the others. At this moment, we have no information about reaction intermediates. Formation of the chlorocyclohexane indicated involvement of the H-abstraction process, giving a cyclohexyl radical in the Tp* and To^M^-supported cobalt catalysts. In the examined conditions, however, the efficiency of the oxidant was limited to around 50% due to concurrent nonproductive degradation of *m*CPBA via radical reaction.

## 3. Materials and Methods

### 3.1. General

Elemental analysis was performed on a Perkin-Elmer CHNS/O Analyzer 2400II (Perkin-Elmer, Waltham, MA, USA). IR spectra were recorded on a FT/IR 4200 spectrometer (JASCO, Tokyo, Japan) for solid samples (as KBr pellets). NMR spectra were recorded on a ECA-600 spectrometer (JEOL, Tokyo, Japan). Magnetic susceptibilities of the cobalt(II) complexes were determined by the Evans method using the ^1^H-NMR spectra of the CDCl_3_ solutions (measured at ambient temperature). UV-vis spectra were measured on a V650 spectrometer (JASCO, Tokyo, Japan). Cyclic voltammetry was performed on a Model 600C Electrochemical Analyzer (ALS, Tokyo, Japan). Gas chromatography (GC) analysis was performed on a GC2010 gas chromatograph (Shimadzu, Kyoto, Japan) equipped with an Rtx-5 column (length = 30 m, i.d. = 0.25 mm, thickness = 0.25 μm, Restek, Bellefonte, PA, USA).

NaTo^M^ [14] and L^Ph^ [16] were prepared according to the literature. All manipulations were performed under argon by standard Schlenk techniques. THF, toluene, CH_2_Cl_2_, and MeCN were purified over a Glass Contour Solvent Dispensing System under Ar atmosphere. *meta*-Chloroperbenzoic acid (*m*CPBA) was washed with KH_2_PO_4_-NaOH buffer solution (pH 7.4) and pure water to remove *meta*-chlorobenzoic acid. Other reagents of the highest grade commercially available were used without further purification.

### 3.2. Preparation and Characterization of the Cobalt(II) Complexes

#### 3.2.1. [Co^II^(Tp*)(acac)] (**1**)

A THF solution (100 mL) of KTp* (1.282 g, 3.812 mmol) was slowly added to a THF solution (50 mL) of Co^II^(acac)_2_ (0.988 g, 3.842 mmol) at room temperature, and the purple slurry solution was stirred for 1 h. The volatile solvent of the solution was evaporated, and the residue was redissolved in CH_2_Cl_2_. The solution was then passed through a filter with Celite to remove any inorganic salts. After evaporation of CH_2_Cl_2_, the residue was redissolved in MeCN and the solution was passed through a filter with Celite. After reduction of solvent volume by evaporation, the solution was subject to recrystallization at −30 °C to give the title complex as deep purple powder (0.731 g, 1.606 mmol, 42.1%). Recrystallization from the MeCN solution at −30 °C gave a pink block crystal suitable for X-ray crystallography.

FT-IR (KBr): *ν*= 3127 (w), 2953 (w), 2924 (vs), 2727 (w), 2504 (vs, *ν*_B-H_), 1591 (vs, *ν*_C=O_), 1520 cm^−1^ (vs, *ν*_C=O_) cm^−1^. UV-vis (CH_2_Cl_2_, r.t.): *λ* = 567 nm (*ε* = 75.7 M^−1^ cm^−1^). UV-vis (MeCN, r.t.): *λ* = 506 nm (*ε* = 39.0 M^−1^ cm^−1^), 567 nm (*ε* = 75.7 M^−1^ cm^−1^). ^1^H-NMR (600 MHz, CDCl_3_, 25 °C, TMS): *δ* = 80.42 (br, 1H; acac-*H*), 64.82 (br, 1H; B-*H*), 55.91 (s, 3H; Pz-*H*_3_), 38.58 (s, 9H; Pz-*CH*_3_), 33.66 (s, 6H; acac-*Me*_2_), −60.44 (br, 9H; Pz-*CH*_3_). ^1^H-NMR (600 MHz, CD_3_CN, 25 °C, TMS): *δ* = 93.22 (br, 1H; acac-*H*), 52.58 (s, 3H; Pz-*H_3_*), 40.55 (s, 9H; Pz-*CH*_3_), 4.64 (s, 6H; acac-*Me*_2_), −69.89 (br, 9H; Pz-*CH*_3_). Magnetic susceptibility: *μ*_eff_ = 4.1 μ_B_. ESI-MS^+^ (MeOH): *m/z* = 478 [[Co^II^(Tp*)(acac)] + Na^+^]^+^. Elemental analysis calcd (%) for [Co^II^(Tp*)(acac)] (C_20_H_29_N_6_BO_2_Co): calc. C 52.77 H 6.42, N 18.46; found: C 52.55, H 6.06, N 18.79.

#### 3.2.2. [Co^II^(To^M^)(acac)] (**2**)

A THF solution (60 mL) of NaTo^M^ (1.329 g, 3.280 mmol) was slowly added to a THF solution (30 mL) of Co^II^(acac)_2_ (0.999 g, 3.886 mmol) at room temperature. The purple slurry solution was stirred for 1 h, and the reaction was heated at reflux for 2 h. The volatile solvent of the solution was evaporated, and the residue was redissolved in CH_2_Cl_2_. The solution was then passed through a filter with Celite to remove any inorganic salts. After evaporation of CH_2_Cl_2_, the residue was redissolved in pentane and the solution was passed through a filter with Celite. After reduction of solvent volume by evaporation, the solution was subject to recrystallization at −30 °C to give the title complex as red purple powder (0.550 g, 1.018 mmol, 31.0%). Recrystallization from the pentane solution at −30 °C for 1 day gave a purple block crystal suitable for X-ray crystallography.

FT/IR (KBr): *ν* = 3077 (w), 3042 (w), 2965 (vs), 2894 (w), 1589 (vs, *ν*_C=O_), 1520 cm^−1^ (vs, *ν*_C=O_). UV-vis (CH_2_Cl_2_, r.t.): *λ* = 450 nm (*ε* = 38.4 M^−1^ cm^−1^), 555 nm (*ε* = 65.4 M^−1^ cm^−1^). UV-vis (MeCN, r.t.): *λ* = 513 nm (*ε* = 123.2 M^−1^ cm^−1^), 557 nm (*ε* = 162.3 M^−1^ cm^−1^), 584 nm (*ε* = 167.6 M^−1^ cm^−1^). ^1^H-NMR (600 MHz, CDCl_3_, 25 °C, TMS): *δ* = 46.69 (br, 1H; acac-*H*), 43.74 (br, 2H; *Ph*), 27.57 (s, 6H; acac-*Me*_2_), 21.57 (s, 2H; *Ph*), 18.04 (s, 1H; *Ph*), 5.81 (s, 6H; Ox-*H*_2_), −60.42 (s, 18H; Ox-*Me*_2_). ^1^H-NMR (600 MHz, CD_3_CN, 25 °C, TMS): *δ* = 41.43 (br, 2H; *Ph*), 40.86 (br, 1H; acac-*H*), 23.83 (s, 6H; acac-*Me*_2_), 20.36 (s, 2H; *Ph*), 17.08 (s, 1H; *Ph*), 5.53 (s, 6H; Ox-*H*_2_), −56.73 (s, 18H; Ox-*Me*_2_). Magnetic susceptibility: *μ*_eff_ = 4.4 μ_B_. ESI-MS^+^ (MeOH): *m/z* = 540 [[Co^II^(To^M^)(acac)] + H^+^]^+^. Elemental analysis calcd (%) for [Co^II^(To^M^)(acac)] (C_26_H_36_N_3_BO_5_Co): calc. C 57.80, H 6.72, N 7.78; found: C 57.46, H 6.68, N 8.02.

#### 3.2.3. [Co^II^(To^M^)(L^Ph^)] (**4**)

An *n*-butyllithium hexane solution (1.64 M, 0.12 mL, 0.197 mmol) was added to a THF solution (20 mL) of HL^Ph^ (0.051 g, 0.192 mmol) at −80 °C to give LiL^Ph^ solution. The solution was then gradually warmed to room temperature with stirring. This LiL^Ph^ solution was slowly added to a THF solution (20 mL) of **2** (0.115 g, 0.213 mmol) at −80 °C. The solution was then gradually warmed to room temperature and stirred for 2 h. The volatile solvent of the solution was evaporated, and the residue was redissolved in CH_2_Cl_2_. The solution was then passed through a filter with Celite to remove any inorganic salts. After evaporation of CH_2_Cl_2_, the red purple solid was washed with pentane to give the title complex as light pink powder (0.089 g, 0.126 mmol, 65.6%). Recrystallization from the Et_2_O/hexane solution at room temperature gave a purple block crystal suitable for X-ray crystallography. We were unable to obtain satisfactory elemental analysis data for this complex.

FT-IR (KBr): *ν* = 3062 (w), 2965 (w), 2927 (vs), 2889 (m), 1602 (w, *ν*_C=N_), 1575 (s, *ν*_C=N_), 1283 cm^−1^ (m, *ν*_BC_). UV-vis (CH_2_Cl_2_, r.t.): λ = 525 nm (*ε* = 441 M^−1^ cm^−1^), 554 nm (*ε* = 580 M^−1^ cm^−1^). Magnetic susceptibility: *μ*_eff_ = 4.0 μ_B_. ESI-MS^+^ (MeOH): *m/z* = 707 [[Co^II^(To^M^)(L^Ph^)] + H^+^]^+^.

#### 3.2.4. [Co^II^(L^Ph^)_2_] (**5**)

An *n*-butyllithium hexane solution (1.55 M, 1.30 mL, 2.015 mmol) was added to a THF solution (25 mL) of HL^Ph^ (0.534 g, 2.006 mmol) at −80 °C to give LiL^Ph^ solution. The solution was then gradually warmed to room temperature with stirring for 1 h. To 10 mL MeOH solution of CoCl_2_ (0.130 g, 1.001 mmol), THF solution of LiL^Ph^ was added, and the resulting reaction mixture was stirred for 1 h. The volatile solvent of the solution was evaporated, and the residue was redissolved in CH_2_Cl_2_. The solution was then passed through a filter with Celite to remove any inorganic salts. After evaporation of CH_2_Cl_2_, the resulting solid was dissolved in MeCN, and refrigeration yielded a pink powder of [Co^II^(L^Ph^)_2_] (0.096 g, 0.163 mmol, 16.3%). Recrystallization from the MeCN solution at room temperature gave a red block crystal suitable for X-ray crystallography.

FT-IR (KBr): *ν* = 3142 (m), 3118 (s), 2917 (vs), 2905 (vs), 2890 (vs), 2836 (vs), 2828 (vs), 2760 (s), 1286 cm^−1^ (vs, *ν*_BC_). UV-vis (CH_2_Cl_2_, r.t.): *λ* = 520 nm (*ε* = 303 M^−1^ cm^−1^), 548 nm (*ε* = 419 M^−1^ cm^−1^). Magnetic susceptibility: *μ*_eff_ = 3.7 μ_B_. Elemental analysis calcd (%) for [Co^II^(L^Ph^)_2_] (C_30_H_36_N_8_B_2_Co): calc. C 61.15, H 6.16, N 19.02; found: C 60.92, H 6.17, N 19.04.

#### 3.2.5. [Co^II^(Tp*)_2_] (**6**)

The previously reported synthetic procedure for **6** [19] was modified as follows. MeOH solution (30 mL) of [Co^II^(OAc)_2_]·4H_2_O (0.927 g, 3.722 mmol) was added to a THF solution (70 mL) of KTp^Me2^ (2.510 g, 7.465 mmol) at room temperature and the yellow solution was stirred for 1.5 h. The volatile solvent of the solution was evaporated, and the residue was redissolved in CH_2_Cl_2_. The solution was then passed through a filter with Celite to remove any inorganic salts. After evaporation of CH_2_Cl_2_, the residue was redissolved in MeCN and the solution was filtered to give the title complex as yellow powder (1.334 g, 2.042 mmol, 54.9%).

FT/IR (KBr): *ν* = 3123 (w), 2976 (w), 2925 (s), 2863 (w), 2733 (s), 2507 (vs, *ν*_B–H_). UV-vis (CH_2_Cl_2_, r.t.): *λ* = 470 nm (*ε* = 130.9 M^−1^ cm^−1^). ^1^H-NMR (600 MHz, CDCl_3_, 25 °C, TMS): *δ* = 45.16 (s, 6H; Pz-*H_3_*), 43.97 (s, 18H; Pz-*CH_3_*), −81.56 (s, 18H; Pz-*CH_3_*). Magnetic susceptibility: *μ*_eff_ = 4.7 μ_B_. ESI-MS^+^ (MeOH): *m/z* = 653 [Co^III^(Tp*)_2_]^+^. Elemental analysis calcd (%) for [Co^II^(Tp*)_2_] (C_30_H_44_N_12_B_2_Co): calc. C 55.15, H 6.79, N 25.73; found: C 54.84, H 6.73, N 25.78.

### 3.3. X-Ray Diffraction Study

Diffraction data for single crystals were collected using a Saturn 70 CDD area detector system (Rigaku, Tokyo, Japan) with graphite monochromated Mo-Kα radiation. The block-shaped crystals were mounted on a CryoLoop with liquid paraffin and flash-cooled to 113 K (for **1**, **2**, and **5**) or 133 K (for **4**) by cold N_2_ gas flow on the goniometer. For complex **1**, three sets of sweeps (at *φ* = 0, 90, and 180°) of data were done using *ω* oscillations from −115.0 to 65.0° in 0.3° steps at *χ* = 45° with an exposure rate of 13.3 s/°, a detector swing angle of −25°, and a crystal-to-detector distance of 55 mm (total of 1800 oscillation images). For complex **2**, two sets of sweeps (at *φ* = 0 and 90°) of data were done using *ω* oscillations from −110.0 to 70.0° in 0.5° steps at *χ* = 45° with an exposure rate of 20 s/°, a detector swing angle of −20°, and a crystal-to-detector distance of 45 mm (total of 720 oscillation images). For complex **4**, two sets of sweeps (at *φ* = 0 and 90°) of data were done using *ω* oscillations from −110.0 to 70.0° in 0.5° steps at *χ* = 45° with an exposure rate of 66.7 s/°, a detector swing angle of −20°, and a crystal-to-detector distance of 45 mm (total of 1200 oscillation images). For **5**, two sets of sweeps (at *φ* = 0 and 90°) of data were done using *ω* oscillations from −110.0 to 70.0° in 0.25° steps at *χ* = 45° with an exposure rate of 80 s/°, a detector swing angle of −20°, and a crystal-to-detector distance of 45 mm (total of 1440 oscillation images).

Data collection and processing were performed using Rigaku CrystalClear software (ver. SM 1.4.0 SP1, Rigaku, Tokyo, Japan) [47]. In all, 22,851 reflections (**1**, *R*_int_ = 0.028), 21,792 reflections (**2**, *R*_int_ = 0.025), 13,756 reflections (**4**, *R*_int_ = 0.050), and 13m918 reflections (**5**, *R*_int_ = 0.139) were collected. Equivalent reflections were merged. The linear absorption coefficient *μ* for Mo-K_α_ radiation was 7.02 cm^−1^ for **1**, 6.05 cm^−1^ for **2**, 5.00 cm^−1^ for **4**, and 5.66 cm^−1^ for **5**. A numerical absorption correction was applied, which resulted in transmission factors ranging from 0.7770 to 0.9077 for **1**, 0.8138 to 0.9041 for **2**, 0.9106 to 0.9708 for **4**, and 0.8349 to 0.9375 for **5**. The data were corrected for Lorentz and polarization effects. Structural solution by a direct method (SIR-92) [48] and refinement by full-matrix least squares (SHELXL-2014/7) [49] against *F*^2^ with all reflections were performed on WinGX software (ver. 2014.1, School of Chemistry, University of Glasgow, Glasgow, UK) [50]. All non-hydrogen atoms were refined anisotropically. Hydrogen atoms adjacent to carbon atoms were placed in calculated positions with C–H = 0.96 Å (for methyl groups) or 0.93Å (for aromatic rings) with Uiso(H) = 1.2 Uiso (attached atom). Hydrogen atoms on boron atoms were located by difference Fourier synthesis and were refined isotropically with B–H = 1.10 Å. The molecular structure was drawn on ORTEP-3 for Windows [51]. Crystal information files (CIFs) of the complexes reported in this paper have been deposited with the Cambridge Crystallographic Data Centre as supplementary publications CCDC-1843690 (**1**) CCDC-1843691 (**2**), CCDC-1843692 (**4**), and CCDC-1893693 (**5**). These data can be obtained free of charge from the Cambridge Crystallographic Data Centre at www.ccdc.cam.ac.uk/data_request/cif. The molecular structures, bond lengths, and angles as well as crystallographic data and structure refinement parameters of **1**, **2**, **4,** and **5** obtained in this work are given in Figure 1 (for **1** and **2**) and Figure 3 (for **4**) and Supplementary Figure S4 (**5**) and Supplementary Table S1.

### 3.4. Catalytic Reactions

#### 3.4.1. Cyclohexene Oxidation with TBHP

A solution of the cobalt(II) compounds (2.0 μmol), Et_3_N (10 μmol), and cyclohexene (2.0 mmol) in MeCN (5 mL) was placed in a Schlenk and degassed with Ar gas. Next, 70% aqueous solution of TBHP (2.0 mmol) was added under Ar atmosphere and stirred at room temperature or 60 °C. The reaction products were analyzed using GC (reaction time 0, 1, 2, 3, 6, 12, 24 h).

#### 3.4.2. Cyclohexane Oxidation with *m*CPBA

A solution of cobalt(II) compounds (2.0 μmol), cyclohexane (15.0 mmol) in MeCN (1 mL), and CH_2_Cl_2_ (1 mL) was placed in a Schlenk and degassed with Ar gas. Next, a CH_2_Cl_2_ (2 mL) solution of *m*CPBA (2.0 mmol) was added under Ar atmosphere and stirred at 35 °C. To monitor product formation, 0.2 mL of the reaction mixture was corrected at certain times (reaction time 0, 10, 30, 60, 90, 120, 180 min) and quenched with a dichloromethane solution (0.5 mL) of triphenylphosphine (10 mg), and then the solution was subjected to GC analysis.

## 4. Conclusions

The coordination behaviors of Tp* and To^M^ toward cobalt(II) with bidentate ligands were compared. Both Tp* and To^M^ coordinate to cobalt(II) with tridentate fashion when the bidentate ligand is the less hindered acetylacetonate. In the bis(1-methylimidazol-2-yl)methylphenylborate (L^Ph^) complexes, Tp* retains the tridentate (*к*^3^) mode, whereas To^M^ functions as the bidentate (*к*^2^) ligand, giving the tetrahedral cobalt(II) complex. The bowl-shaped cavity derived from the six methyl groups on To^M^ leads to susceptibility to the bulkiness of the opposite bidentate ligand. The entitled scorpionate compounds catalyze degradation of TBHP to induce the peroxidation of cyclohexene, although their catalytic efficiencies are lower than those of Co(OAc)_2_ and Co(acac)_2_. On the cyclohexane oxidation with *m*CPBA, both To^M^ and Tp* complexes function as the catalysts. The difference in the electron-donating abilities of the scorpionates, that is, To^M^ > Tp*, is reflected in the initial reaction rates.

## Figures and Tables

**Table 1:** Cyclohexene oxidation with *tert*-butyl hydroperoxide (TBHP) at room temperature. 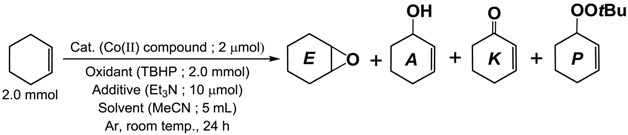

Catalyst (Cat.)	Products/μmol				TON *^1^
	*E*	*A*	*K*	*P*	
[Co^II^(Tp*)(acac)] (**1**)	0.5	6.4	20.4	52.7	50.2
[Co^II^(To^M^)(acac)] (**2**)	1.3	12.1	49.4	85.2	98.7
[Co^II^(Tp*)(L^Ph^)] (**3**)	0.7	1.9	12.1	13.3	20.0
[Co^II^(To^M^)(L^Ph^)] (**4**)	1.1	5.0	24.7	73.9	64.7
[Co^II^(L^Ph^)_2_] (**5**)	0.7	6.9	27.5	23.0	42.8
[Co^II^(Tp*)_2_] (**6**)	0.7	5.8	28.1	70.2	66.4
none	0.0	0.4	4.6	2.7	−
Co^II^(acac)_2_·2H_2_O	1.2	7.7	21.9	187.7	120.2
Co^II^(OAc)_2_·4H_2_O	1.1	10.0	27.0	195.0	130.1

*^1^ TON = (cyclohexene oxide (***E***) + cyclohexen-1-ol (***A***) + cyclohexen-1-one (***K***) × 2 + 3-(*tert*-butylperoxy)-1-cyclohexene (***P***))/cobalt(II) compound.

**Table 2:** Cyclohexene oxidation with TBHP at 60 °C. 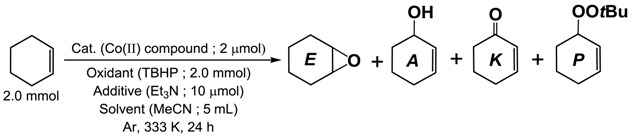

Catalyst (Cat.)	Products/μmol				TON *^1^
	*E*	*A*	*K*	*P*	
[Co^II^(Tp*)(acac)] (**1**)	3.0	11.9	32.8	345.2	212.9
[Co^II^(To^M^)(acac)] (**2**)	6.4	5.6	49.5	702.6	406.8
[Co^II^(Tp*)(L^Ph^)] (**3**)	1.7	4.0	33.6	117.7	95.3
[Co^II^(To^M^)(L^Ph^)] (**4**)	4.9	5.0	56.1	693.2	407.7
[Co^II^(L^Ph^)_2_] (**5**)	1.4	6.4	27.0	105.3	83.5
[Co^II^(Tp*)_2_] (**6**)	3.6	3.6	36.1	612.4	345.9
none	1.1	5.9	26.8	95.2	−
Co^II^(acac)_2_·2H_2_O	6.4	10.2	84.2	871.1	528.1
Co^II^(OAc)_2_·4H_2_O	6.3	28.1	118.5	735.6	503.5

*^1^ TON = (cyclohexene oxide (***E***) + cyclohexen-1-ol (***A***) + cyclohexen-1-one (***K***) × 2 + 3-(*tert*-butylperoxy)-1-cyclohexene (***P***))/cobalt(II) compound.

**Table 3:** Cyclohexane oxidation with *meta*-chloroperbenzoic acid (*m*CPBA) at 35 °C. 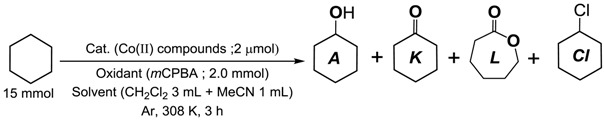

Catalyst (Cat.)	Products/μmol				TON *^1^	*A*/(*K* + *L*) *^2^
	*A*	*K*	*L*	*Cl*		
[Co^II^(Tp*)(acac)] (**1**)	587.2	104.1	52.4	36.2	468.1	3.8
[Co^II^(To^M^)(acac)] (**2**)	640.0	154.8	32.5	47.8	531.2	3.4
[Co^II^(Tp*)(L^Ph^)] (**3**)	705.2	133.6	48.1	58.1	563.3	3.9
[Co^II^(To^M^)(L^Ph^)] (**4**)	605.4	108.8	36.7	54.8	475.6	4.2
[Co^II^(L^Ph^)_2_] (**5**)	630.3	115.9	37.0	55.5	495.8	4.1
[Co^II^(Tp*)_2_] (**6**)	641.8	125.4	32.9	50.3	504.3	4.1
none	69.4	0.5	2.1	3.8	−	26.6
Co^II^(acac)_2_·2H_2_O	456.2	146.6	18.4	26.7	406.5	2.8
Co^II^(OAc)_2_·4H_2_O	609.9	181.3	33.2	33.1	535.9	2.8

*^1^ TON = {cyclohexanol (***A***) + chlorocyclohexane (***Cl***) + 2 × {cyclohexanone (***K***) + *ε*-caprolactone (***L***)}}/cobalt(II) compound. *^2^ Alcohol selectivity = (cyclohexanol (***A***))/(cyclohexanone (***K***) + *ε*-caprolactone (***L***)).

## Supplementary Materials

The following are available online. Figure S1: ^1^H-NMR spectra of CDCl_3_ and CD_3_CN solutions of **1**. Figure S2: ^1^H-NMR spectra of CDCl_3_ and CD_3_CN solutions of **2**. Figure S3: FT/IR spectra of KBr pellets of **1**, **2**, **4**, and **5** measured at room temperature. Figure S4: UV-vis spectra of CH_2_Cl_2_ solutions of **4** and **5**. Figure S5: Molecular structure of **5**. Figure S6: Cyclic voltammograms of **1**–**5**. Figure S7: Time course of cyclohexene oxidation with TBHP mediated by **1**–**6**, Co^II^(acac)_2_·2H_2_O, and Co^II^(OAc)_2_·4H_2_O. Figure S8: Product analysis for cyclohexene oxidation with TBHP by **2** with or without the PPh_3_ quencher. Table S1: Crystallographic data of **1**, **2**, **4,** and **5**.
