# Supplementary material for: Cobalt(II) Complexes with N,N,N-Scorpionates and Bidentate Ligands: Comparison of Hydrotris(3,5-dimethylpyrazol-1-yl)borate Tp* vs. Phenyltris(4,4-dimethyloxazolin-2-yl)borate ToM to Control the Structural Properties and Reactivities of Cobalt Centers
Source: Molecules. 2018 Jun 16;23(6):1466. doi: 10.3390/molecules23061466 (PMC6099786; doi:10.3390/molecules23061466)
Supplement: Supplementary file 1 [file molecules-23-01466-s001.pdf]

*Supporting Information*

# Cobalt(II) Complexes with *N,N,N*-Scorpionates and Bidentate Ligands: Comparison of Hydrotris(3,5-dimethylpyrazol-1-yl)borate Tp* vs. Phenyltris(4,4-dimethyloxazolin-2-yl)borate To$^{M}$ to Control the Structural Properties and Reactivities of Cobalt Centers

**Toshiki Nishiura, Takahiro Uramoto, Yuichiro Takiyama, Jun Nakazawa * and Shiro Hikichi ***

Department of Material and Life Chemistry, Faculty of Engineering, Kanagawa University, 3-27-1 Rokkakubashi, Kanagawa-ku, Yokohama 221-8686, Japan

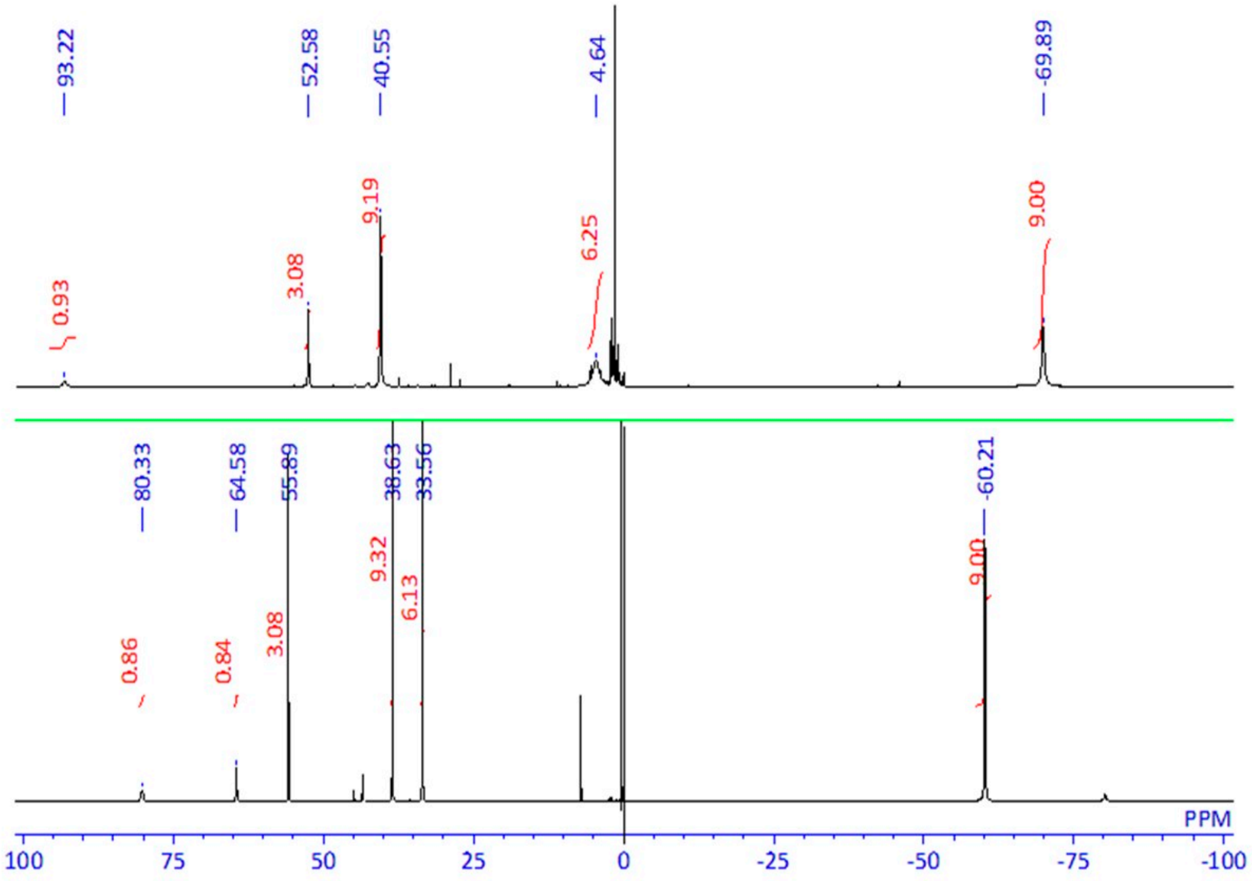


**Figure S1.** $^{1}$H-NMR spectra of the $CD_3CN$ (**top**) and $CDCl_3$ (**bottom**) solutions of [Co$^{II}$(acac)(Tp*)] (**1**) (600 MHz; measured at ambient temperature).

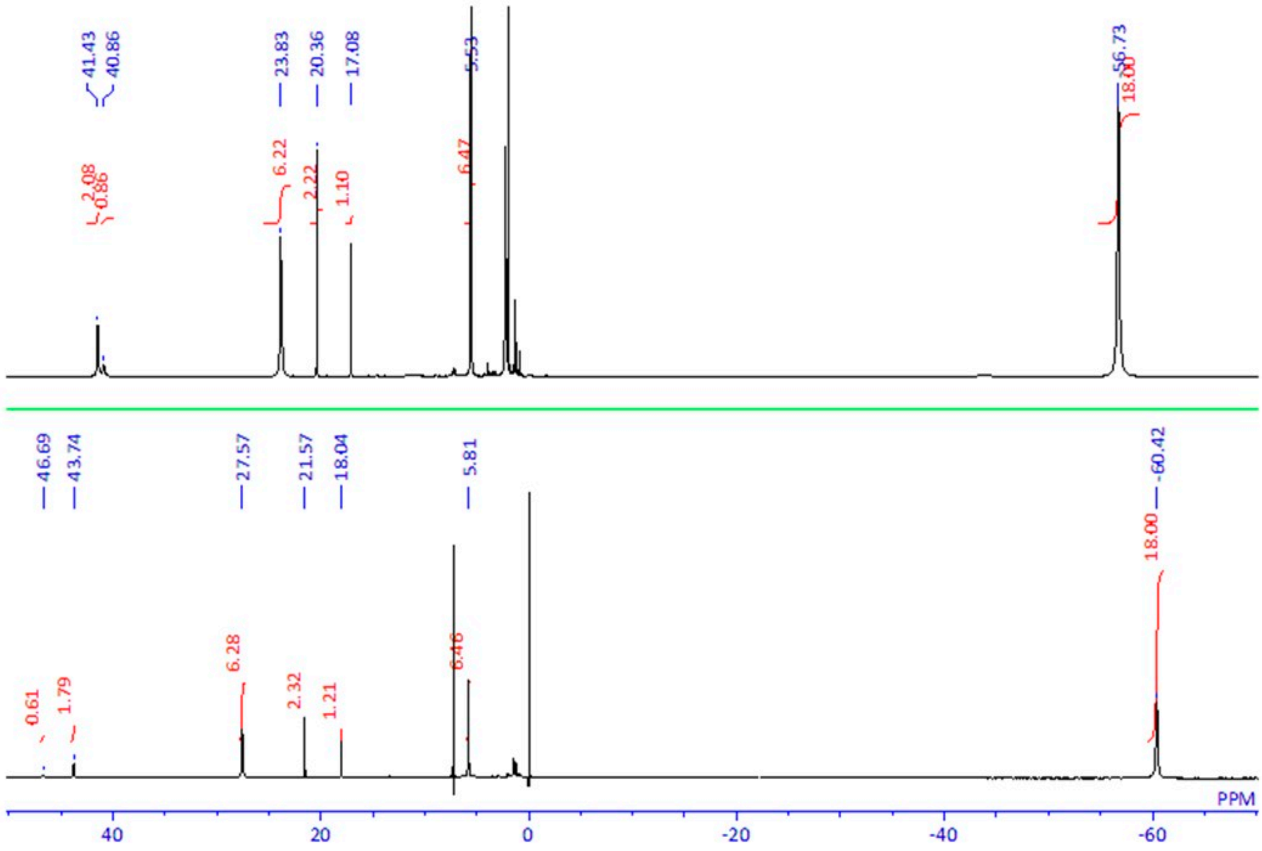


**Figure S2.** $^{1}$H-NMR spectra of the $CD_3CN$ (**top**) and $CDCl_3$ (**bottom**) solutions of [Co$^{II}$(acac)(To$^M$)] (**2**) (600 MHz; measured at ambient temperature).

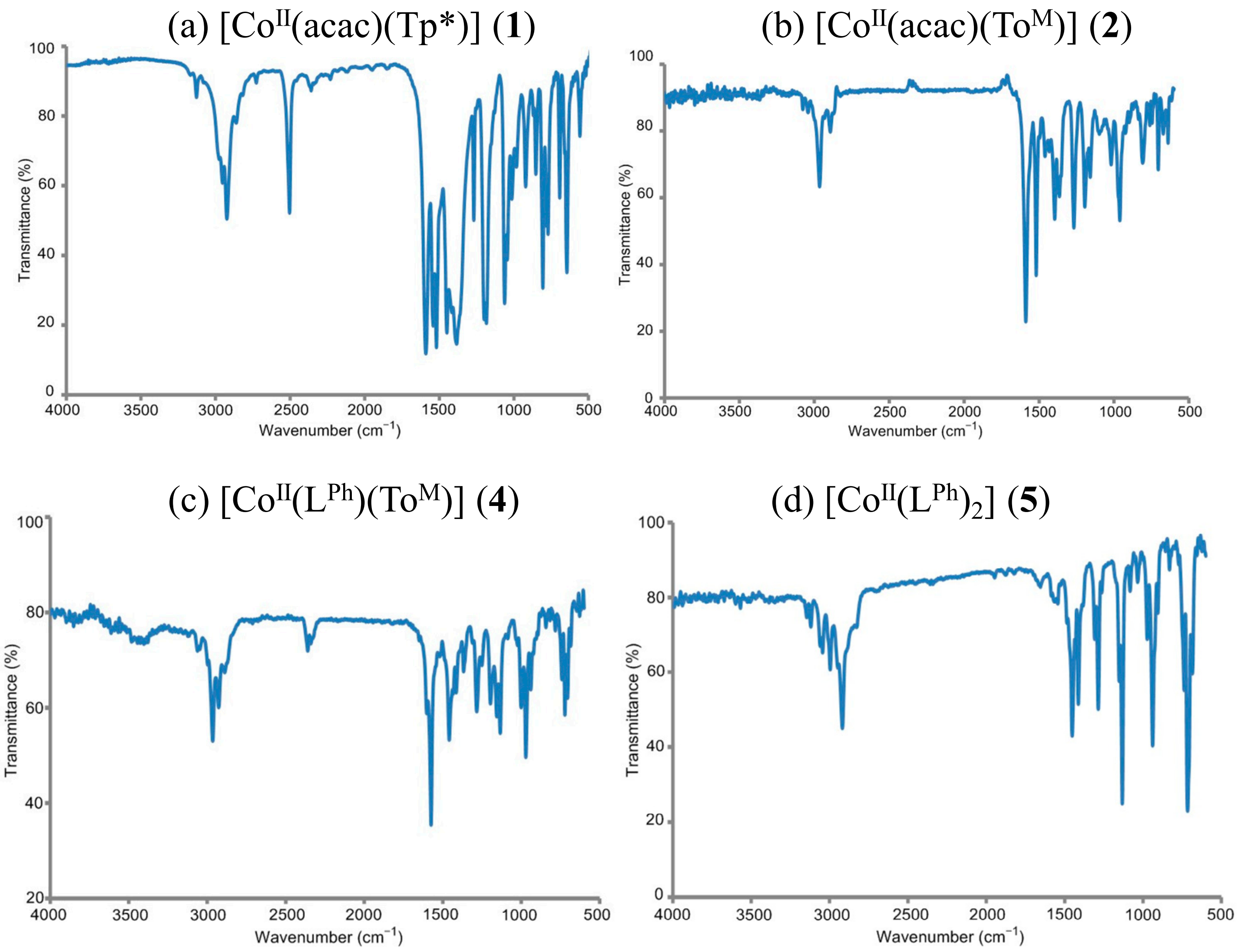


**Figure S3.** FT/IR spectra of KBr pellets of [$Co^{II}$(acac)(Tp*)] (**1**; (**a**)), [$Co^{II}$(acac)($To^{M}$)] (**2**; (**b**)), [$Co^{II}$($L^{Ph}$)($To^{M}$)] (**4**; (**c**)) and [$Co^{II}$($L^{Ph}$)$_2$] (**5**; (**d**)) measured at room temperature.

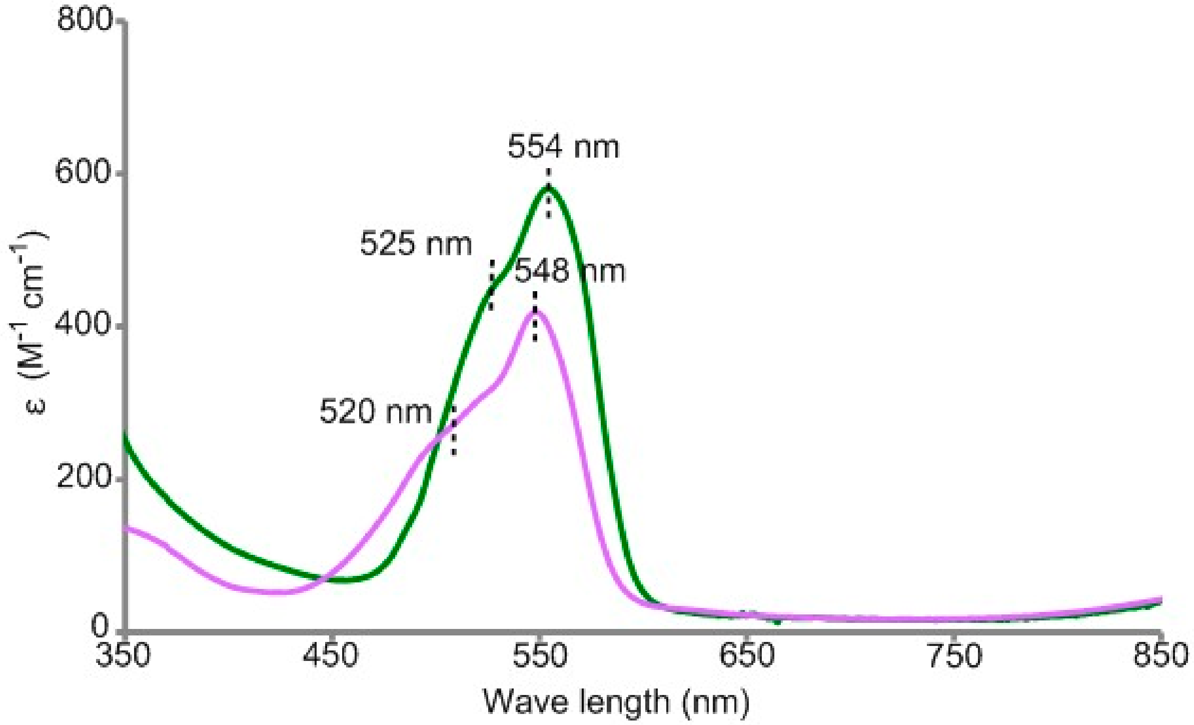


**Figure S4.** UV-vis spectra of the $CH_2Cl_2$ solutions of $[Co^{II}(To^M)(L^{Ph})_2]$ (**4** ; green) and$[Co^{II}(L^{Ph})_2]$ (**5** ; purple) measured at room temperature.

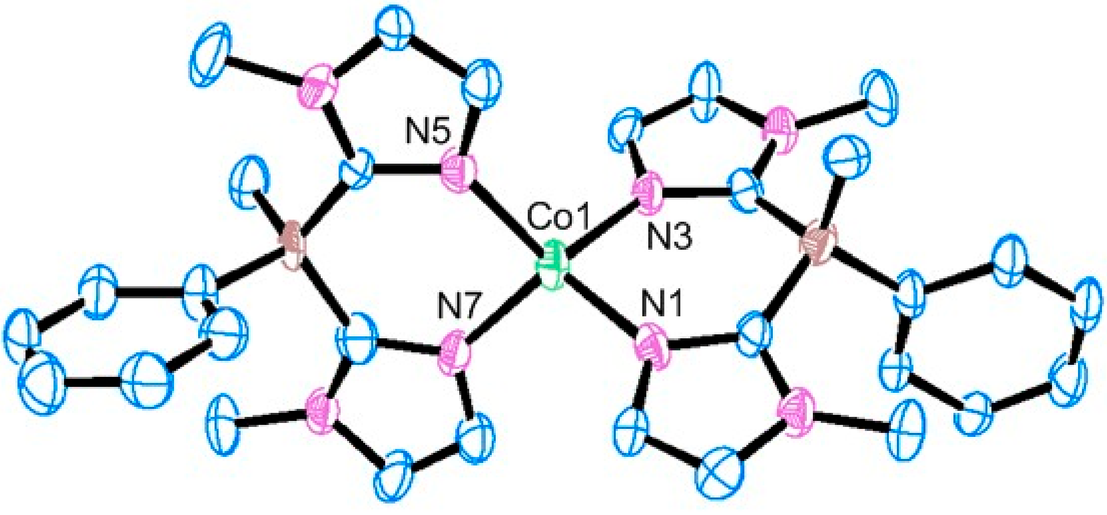


**Figure S5.** Molecular structure of **5**. All hydrogen atoms are omitted for clarity; thermal ellipsoids are set at 30% probability. Selected bond lengths [Å] and angles [°]: Co1-N1 1.997 (8), Co1-N3 1.992 (8), Co1-N5 1.996 (8), Co1-N7 1.994 (8), N1-Co1-N3 95.8 (3), N1-Co1-N5 125.5 (3), N1-Co1-N7 108.2 (3), N3-Co1-N5 109.3 (3), N3-Co1-N7 123.7 (3), N5-Co1-N7 96.9 (3).

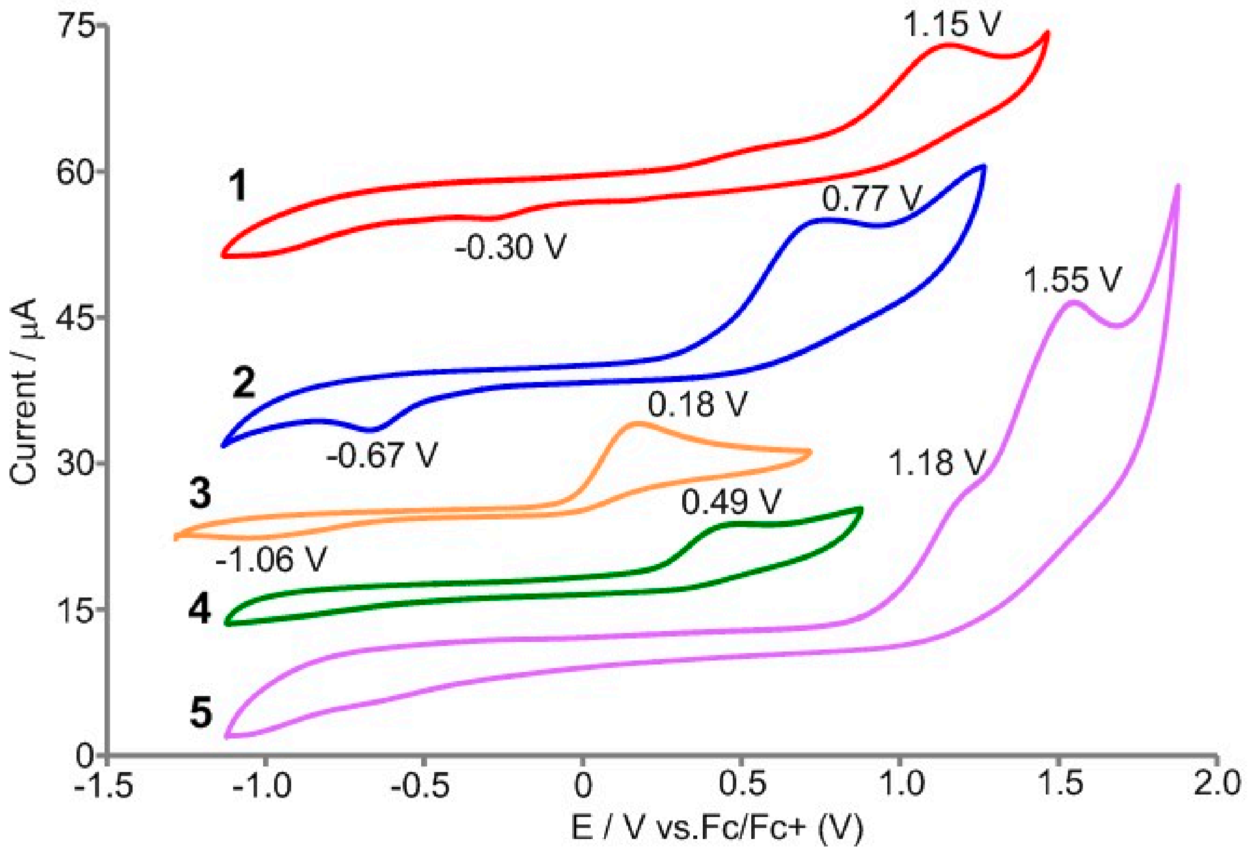


**Figure S6.** Cyclic voltammograms of **1**–**5**. Measurement conditions: working electrode; Pt disc, counter electrode; Pt wire, reference electrode; Ag/AgCl, amount of cobalt(II) complexes; 5 μmol, solvent; MeCN (for **1**, **2**, **4**, **5**) and $CH_2Cl_2$ (for **3**), supporting electrolyte; $(^{n}Bu_4N)PF_6$ 0.1 M, atmosphere; Ar, temperature; ambient temp. scan rate : 100 mV/s.

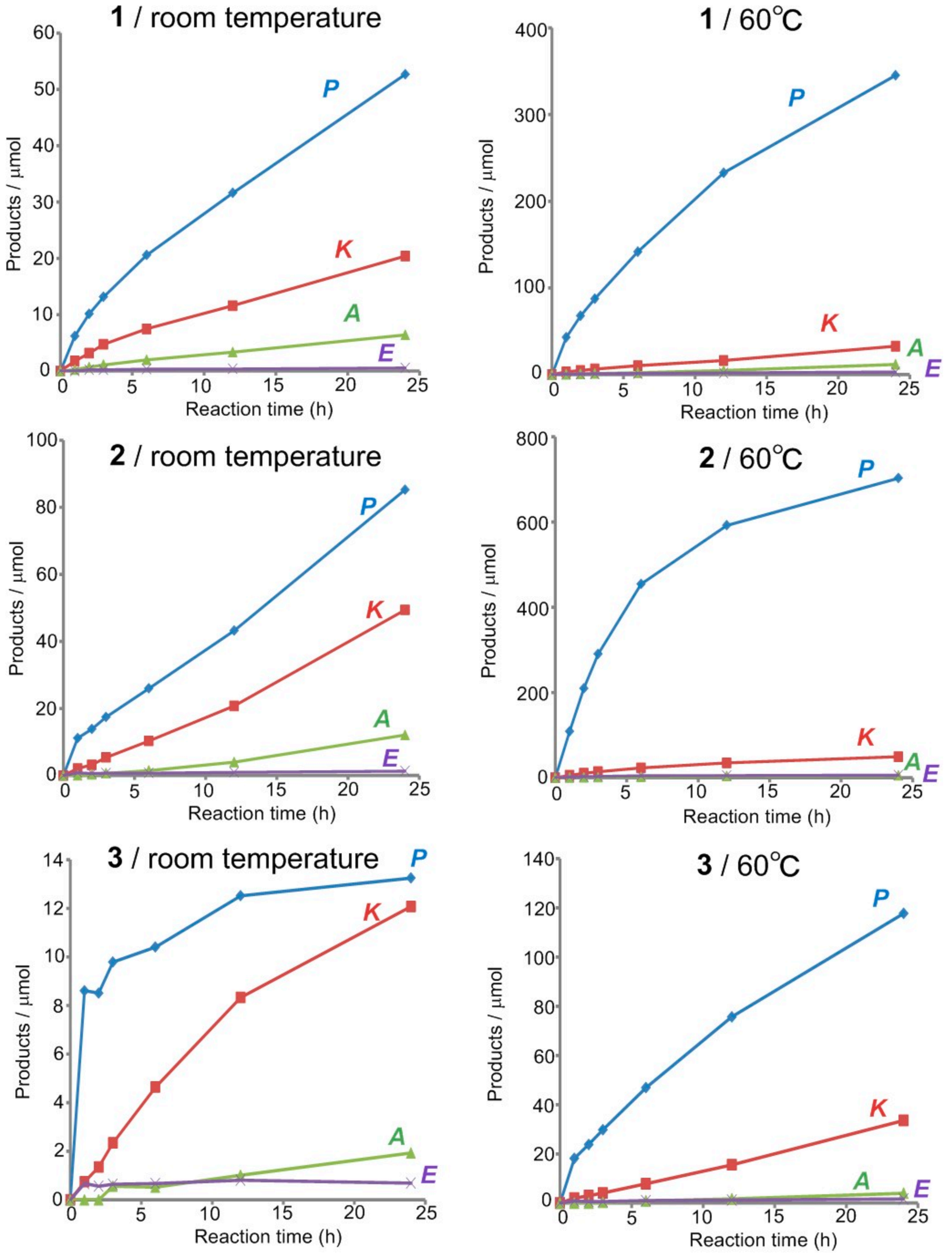


**Figure S7.** Time course of cyclohexene oxidation with TBHP mediated by **1–6**, $Co^{II}(acac)_2{\cdot}2H_2O$ and $Co^{II}(OAc)_2{\cdot}4H_2O$.

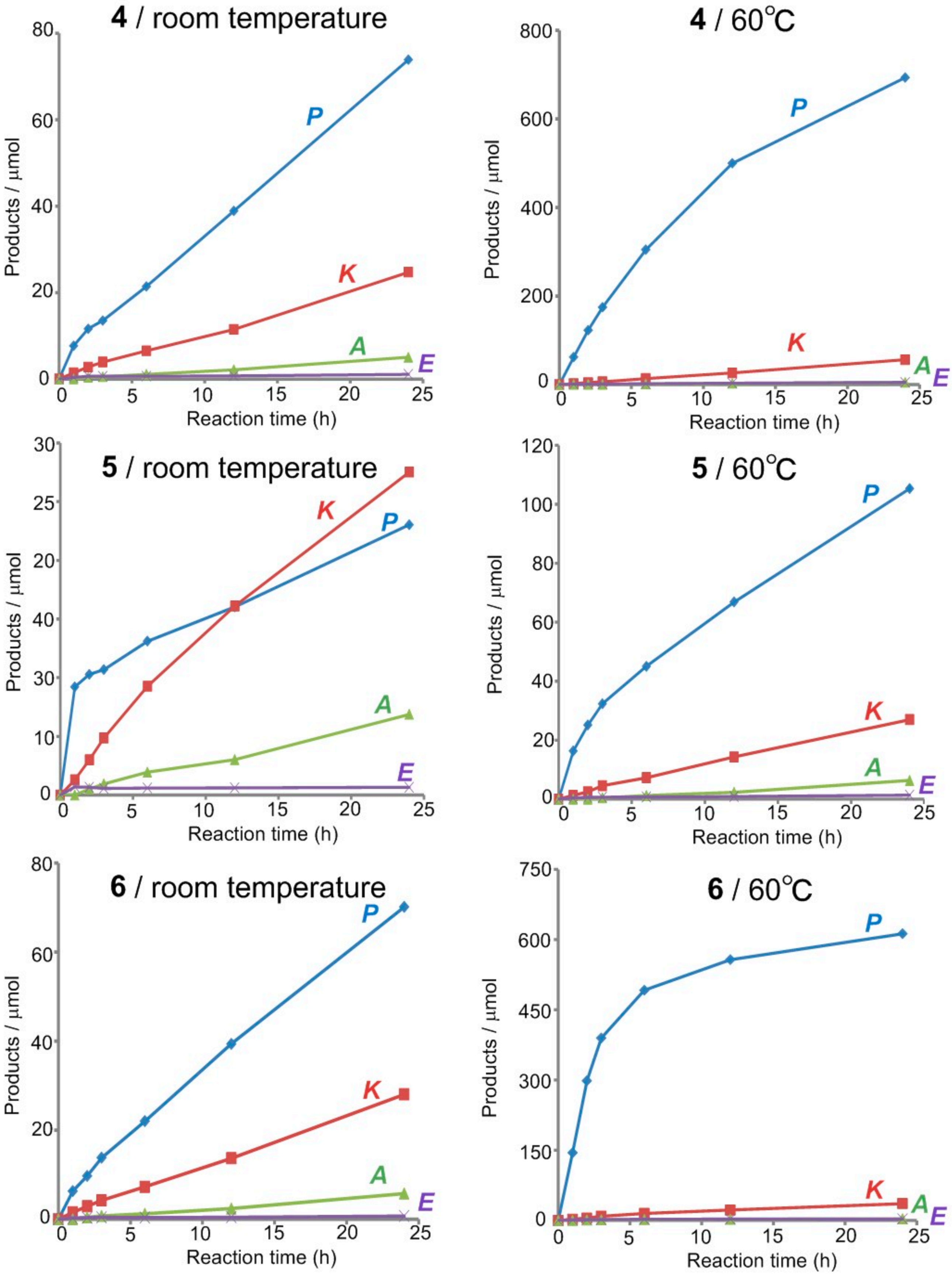


**Figure S7.** (continued)

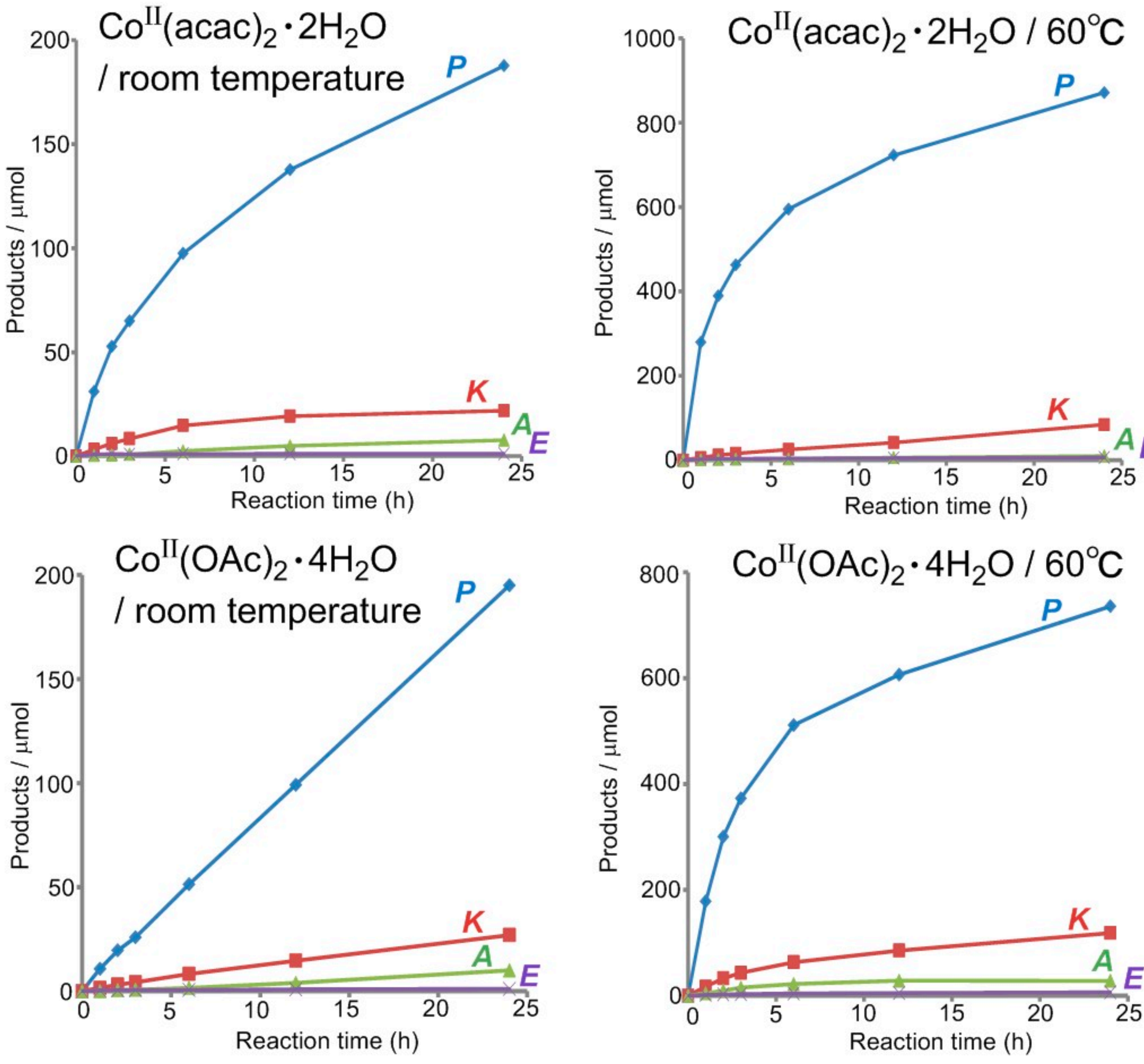


Figure **S7.** (continued)

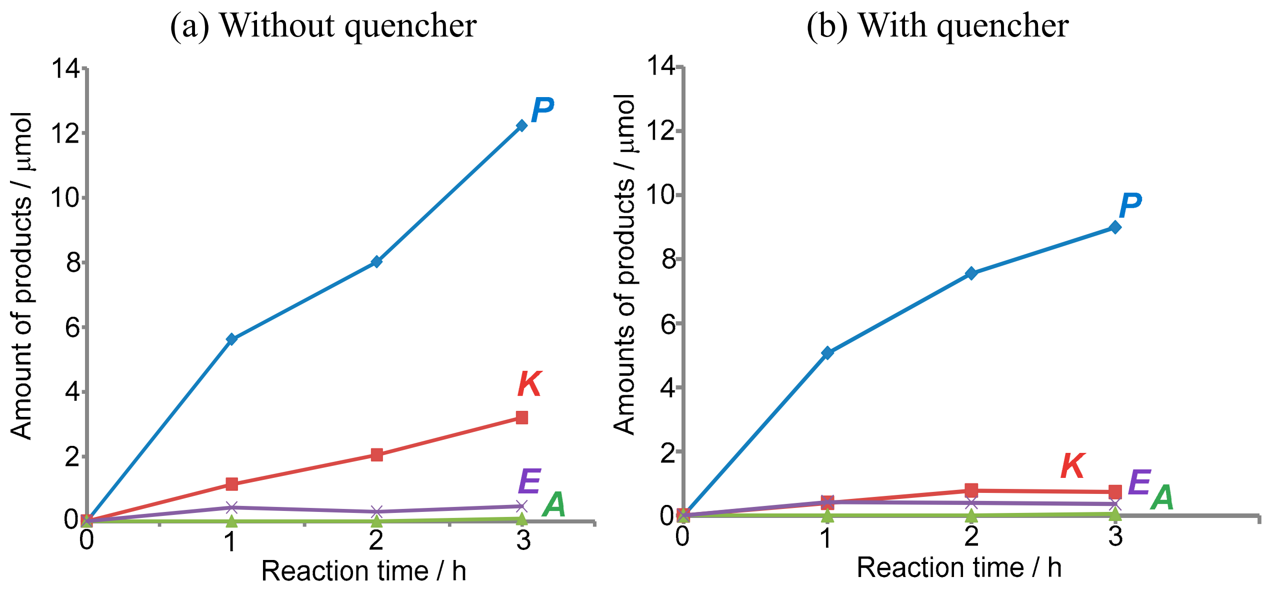


**Figure S8.** Products analysis for the cyclohexene oxidation with TBHP by **2** at ambient temperature with or without of the $PPh_3$ quencher.

**Table S1.** Crystallographic data and structure refinement parameters for **1**, **2**, **4** and **5**.

| Complex | **1**·MeCN | **2**·(pentane)$_{0.5}$ | **4** | **5** |
|---|---|---|---|---|
| Formula | $C_{22}H_{32}BCoN_7O_2$ | $C_{26}H_{36}BCoN_3O_5$·$(C_5H_{12})_{0.5}$ | $C_{36}H_{47}B_2CoN_7O_3$ | $C_{30}H_{36}B_2CoN_8$ |
| Formula weight | 496.28 | 576.41 | 706.35 | 589.22 |
| Crystal color, habit | Pink, Block | Purple, Block | Purple, Block | Red, Block |
| Size/mm | 0.20, 0.30, 0.40 | 0.20, 0.20, 0.50 | 0.05, 0.15, 0.25 | 0.05, 0.25, 0.35 |
| Crystal system | Monoclinic | Monoclinic | Triclinic | Monoclinic |
| Space group | $P2_1/n$ (#14) | $P2_1/n$ (#14) | P-1 (#2) | $P2_1/n$ (#14) |
| $a$/Å | 11.2775(6) | 9.006(3) | 11.039(8) | 19.87(2) |
| $b$/Å | 19.0252(9) | 22.243(7) | 11.658(8) | 7.585(9) |
| $c$/Å | 12.1374(9) | 15.742(5) | 14.845(11) | 21.65(3) |
| $\alpha$/deg | 90 | 90 | 80.14(2) | 90 |
| $\beta$/deg | 100.542(3) | 105.644(4) | 89.58(2) | 100.02(2) |
| $\gamma$/deg | 90 | 90 | 88.50(3) | 90 |
| $V$/Å$^3$ | 2260.2(2) | 3036.6(4) | 1881.5(7) | 3213(7) |
| $Z$ | 4 | 4 | 2 | 4 |
| $F$(000) | 1044 | 1224 | 746 | 1236 |
| $D$(calcd)/g·cm$^{-3}$ | 1.288 | 1.261 | 1.247 | 1.218 |
| $\mu$ (Mo-K$\alpha$) cm$^{-1}$ | 7.02 | 6.05 | 5.00 | 5.66 |
| Temp./K | 113 | 133 | 113 | 113 |
| Unique reflections | 5843 | 6694 | 7821 | 6469 |
| Observed reflections $I > 2\sigma(I)$ | 5291 | 5746 | 4858 | 1160 |
| Parameter refined | 311 | 438 | 452 | 376 |
| $2\theta_{max}$/deg | 55.0 | 55.0 | 54.6 | 54.0 |
| $R$($I$ >2$\sigma$($I$), all)[a] | 0.0338, 0.0388 | 0.0314, 0.0374 | 0.0682, 0.1051 | 0.120, 0.247 |
| $R_w$($I$ >2$\sigma$($I$), all)[a] | 0.0815, 0.0846 | 0.0815, 0.0849 | 0.1684, 0.1992 | 0.270, 0.365 |
| Goodness of fit $S$[b] | 1.061 | 1.045 | 1.022 | 0.784 |

[a] $R = \sum||F_o| - |F_c|| / \sum|F_o|$. $R_w = \{\sum[w(F_o^2 - F_c^2)^2] / \sum[w(F_o^2)^2]\}^{1/2}$. [b] $S = \{\sum[w(F_o^2 - F_c^2)^2] / (n-p)\}^{1/2}$, where $n$ is the number of reflections and $p$ is the total number of parameters refined.
